# COVID-19-Related Stressors and Chinese Adolescents' Adjustment: The Moderating Role of Coping and Online Learning Satisfaction

**DOI:** 10.3389/fpsyt.2021.633523

**Published:** 2021-03-30

**Authors:** Xiaoshan Li, Xiujuan Tang, Hou Wu, Pengyong Sun, Min Wang, Li Li

**Affiliations:** ^1^School of Psychology, Jiangxi Normal University, Nanchang, China; ^2^Center of Mental Health Education and Research, School of Psychology, Jiangxi Normal University, Nanchang, China; ^3^Nanchang Institute of Technology, Nanchang, China

**Keywords:** COVID-19-related stressors, adjustment, coping, online learning satisfaction, adolescent

## Abstract

The present study aims to examine the main and interactive relations of COVID-19-related stressors, coping, and online learning satisfaction with Chinese adolescents' adjustment during the COVID-19 pandemic. A total of 850 adolescents from three Chinese secondary schools participated in the survey during the pandemic outbreak, and the data were analyzed by hierarchical linear regression. The results show that COVID-19-related stressors were a vulnerability factor in predicting adjustment. Adolescents' adjustment could be attributed to both individual-level (e.g., coping) and class-level (e.g., a class-level indicator of coping) characteristics. Specifically, problem-based coping and online learning satisfaction can promote adolescents' adjustment directly or serve as a buffer against the negative impact of stressors on adjustment, while emotion-based coping is a vulnerability factor in predicting adjustment directly or as a risk factor in strengthening the relation between stressors and adjustment. Compared with male adolescents and adolescents with high socio-economic status, female and impoverished adolescents reported poorer adjustment during the COVID-19 pandemic. These findings enrich our understanding of the impact of the COVID-19 pandemic on adolescents' adjustment and are helpful in improving adolescents' adjustment during the pandemic.

## Introduction

The COVID-19 pandemic has caused great suffering for people living in infected areas, and people have developed many adjustment problems (e.g., emotional problems, physical health problems) due to the high infection and mortality rates, which cannot yet be prevented by a vaccine (1, 2). Specifically, adolescents are susceptible to stressful events due to brain and body immaturity and may develop more adjustment problems (2–4). However, recent studies on the COVID-19 pandemic have focused mainly on its impact on patients and health care staff rather than on the general adolescent population (5, 6). In addition, nearly all adolescents on the Chinese mainland were asked to take online courses at the new semester in 2020 due to the pandemic. As for the adolescents, online learning is also a new life event. However, we know little by far about how the online learning experience influences adolescents' adjustment. Therefore, the present study aims to investigate the relation between COVID-19-related stressors and adolescents' adjustment, and the possible moderators between them.

### COVID-19-Related Stressors and Adolescents' Adjustment

Many factors can influence adolescents' adjustment, and stress is one of the most common concepts in the health literature. According to Lazarus and Folkman (7), stressors are those events that might endanger well-being. The negative impact of stressors (including stressors related to infectious disease) on adolescents' adjustment has been well-documented in the literature (8, 9). During the COVID-19 pandemic, adolescents have engaged in fewer activities than before. For example, their vocational plans have been canceled, no massive gatherings have been allowed, and attending courses online has become their main activity. These issues could be stressors that are harmful to their physical or mental health (2, 6, 10–12). Therefore, adolescents who experience more COVID-19-related stressors might report poorer adjustment than those who experience fewer.

### The Role of Coping in Predicting Adolescents' Adjustment

Coping is often regarded as a moderator in discussing stress-health relations. According to Lazarus and Folkman (7), coping refers to individuals' constantly changing their cognitive and behavioral efforts in dealing with challenging physical or environmental situations. There are two prominent types of coping strategies in the literature: problem-based coping (e.g., managing the problem) and emotion-based coping (e.g., regulating the emotional response to the problem). When individuals engage in emotion-based coping, the threatening situation is not changed at its source, so the stress is only temporarily ameliorated, and psychological problems often emerge or become more complex over time (8). In contrast, when individuals engage in problem-based coping, they tend to develop a better understanding of the problem through cognitive restructuring and/or engaging in problem solving (e.g., seeking medical treatment) to change the threatening situation, which could effectively reduce the negative effect of stressors on adjustment (7). Research has shown that emotion-focused coping is a risk factor in increasing the negative impact of stressors on adjustment, while problem-focused coping is regarded as a buffer in reducing the negative impact of stressors on adjustment (8, 9). Thus, the present study hypothesized that problem-based coping can reduce the negative effects of COVID-19-related stressors on adjustment, while emotion-based coping can amplify the negative impact of COVID-19-related stressors on adjustment.

### The Role of Online Courses in Predicting Adolescents' Adjustment

For most adolescents, especially Asian adolescents, academic achievement is a means not only of attaining personal aspirations but also of fulfilling the expectations of parents, teachers, and significant others (13). Failing to achieve academic goals can lead to feelings of futility and despondency, which may produce susceptibility to psychological symptoms such as depression (14). During the pandemic, online learning satisfaction (referring to perceived satisfaction with online courses) (15) has been one of the most important indicators of academic achievement. Adolescents with higher online learning satisfaction tend to have a high sense of control and pursue problem-based coping in stressful conditions, which can reduce the negative impact of stressors on their adjustment. Therefore, online learning satisfaction might be regarded as a buffer in the relation between COVID-19-related stressors and adjustment.

### The Present Study

The objective of the present study was to examine the relationship between COVID-19-related stressors and Chinese adolescents' adjustment during the pandemic and the possible moderating role of coping and online learning satisfaction. In addition, previous research has shown that individual adjustment (e.g., psychological symptoms) can be influenced by the class or school atmosphere (16, 17). During the pandemic, adolescents have participated in different online class groups (e.g., WeChat groups). Students within one class learn and communicate together, and each class forms its own classroom climate in dealing with stressors related to the COVID-19 pandemic through the interactions of teachers and students. Therefore, the impact of class-level coping or online learning satisfaction on adjustment was also explored in the present study.

## Methods and Materials

### Participants

With the written informed consent of their parents or guardians, a total of 850 students from three Chinese secondary schools participated in the survey in April 2020 (after 1 month of taking online courses). With the help of head teachers, all participants received an email asking them to complete an anonymous questionnaire independently and return it by the deadline. The survey included indicators of adjustment (e.g., anxiety), COVID-19-related stressors, coping, online learning satisfaction, and demographic variables. The response rate was 96%. Questionnaires with more than 15% of the items unanswered were excluded from the later analysis. A total of 802 secondary school students from 29 classes completed the survey, and their data were used in the following analysis. In the present sample, 425 (53%) of the students were female. There were 435 (54.2%) students in grade seven (*M*_age_ = 12.95, SD = 0.58), 189 (23.6%) students in grade eight (*M*_age_ = 13.79, SD = 0.71) and 178 (22.2%) students in grade nine (*M*_age_ = 14.72, SD = 0.60). The present study was conducted under the approval of the moral and ethical committee of the School of Psychology, Jiangxi Normal University.

### Instruments

#### COVID-19-Related Stressors

The COVID-19-related stressors checklist (18) was used to assess the participants' experience of COVID-19-related stressors. The checklist consisted of 16 COVID-19-related stressors that were generalized into the following six groups: self-related events, family-related events, friend-related events, acquaintance-related events, information-related events, and other infectious disease-related events. The participants were asked to report whether they had experienced COVID-19-related stressors such as “canceling a vocational trip due to the COVID-19 pandemic.” A score of 1 indicates that the participants had experienced COVID-19-related stressors during the pandemic, while a score of 0 indicates that participants had not experienced any. The total number of events endorsed across all categories was computed, and a high score indicated adolescents who experienced more COVID-19-related stressors.

##### Adjustment

Three indicators were used to assess individual adjustment: anxiety, depression, and perceived general health. The GAT-7 (19) and PHQ-9 (20) were used to measure adolescents' symptoms of anxiety and depression, respectively. The participants responded on a four-point scale (0 = not at all, 3 = nearly every day), with a higher score indicating higher symptoms of anxiety or depression. The Cronbach αs of the GAD-7 and PHQ-9 were 0.91 and 0.87 in the present study, respectively. For perceived general health, a single-item self-rating of perceived health (“Overall, your health status is___”) (8) was used. The participants responded on a 5-point scale (1 = poor, 4 = excellent), with a higher score indicating good health.

##### Moderators

In the present study, two moderators were used to discuss the relationship between COVID-19-related stressors and adolescents' adjustment on the one hand and coping and online learning satisfaction on the other. The brief coping strategy scale (21) was used to measure the frequency of participants' use of problem-based coping (e.g., active coping) and emotion-based coping (e.g., denying) during the COVID-19 pandemic. The participants responded to each item on a four-point scale (1 = never used, 4 = always used). The Cronbach αs of problem-based coping and emotion-based coping were 0.86 and 0.79 in the present study, respectively. Online learning satisfaction was measured by one item, “To assess perceived satisfaction with individual online courses during the pandemic,” with responses on a scale between 1 (very dissatisfied) and 5 (very satisfied).

##### Control variables

Previous research showed that some demographic variables (e.g., gender, social-economic status) can predict individual adjustment during the pandemic (18). Therefore, both variables of gender and socioeconomic status were used as control variables in present study. Socioeconomic status was measured by the sum of the parents' education level and the family income. The parents' education level ranged from 1 (primary school or below) to 5 (bachelor's degree or above), and the annual family income ranged from 1 (<20,000 Yuan) to 6 (more than 200,000 Yuan). Socioeconomic status was divided into three ranks: low (-1 SD from the mean), middle (a score between−1 SD from the mean and + 1 SD from the mean) and high (+ 1 SD from the mean).

#### Data Analysis

The data were analyzed using SPSS 16. First, a descriptive statistical analysis was conducted. Second, the adolescents in the present study were nested within each class, indicating that the database had a hierarchical structure. A hierarchical linear regression model was used to investigate the relations among the variables studied, with anxiety, depression, and perceived general health as outcome variables and COVID-19-related stressors, coping, online learning satisfaction, and demographic variables as predictors. The variables of coping, online learning satisfaction, and demographic factors (e.g., gender) were regarded as individual-level variables, while the mean scores of students' coping and online learning satisfaction were regarded as class-level variables. One-way ANOVA showed that only the demographic variables of gender and socioeconomic status significantly predicted an individual's adjustment. Therefore, gender and socioeconomic status were included as covariates in the final equation. Coping and online learning satisfaction were mean-centered, as suggested by Aiken and West (22). Multicollinearity was not considered a problem because the variance in inflation factors for all terms in the models did not exceed the cutoff of 7 (22).

## Results

### Descriptive Analysis

The means, standard deviations, and zero-order correlations for the full sample are presented in Table 1. The results showed that all constructs were closely related to each other, with the exception of the relationship between perceived general health and emotion-based coping.

**Table 1:** Descriptive and correlative analysis of study variables.

**Variable**	**M ± SD**	**1**	**2**	**3**	**4**	**5**	**6**	**7**
1. Anxiety	9.24 ± 6.14	1						
2. Depression	11.4 ± 7.58	0.76^**^	1					
3. Perceived general health	4.02 ± 0.96	−0.17^**^	−0.17^**^	1				
4. Problem-based coping	30.3 ± 8.54	−0.15^**^	−0.13^**^	0.17^**^	1			
5. Emotion-based coping	14.65 ± 6.0	0.45^**^	0.50^**^	0.03	0.48^**^	1		
6. COVID-19-related stressors	2.04 ± 1.29	0.32^**^	0.33^**^	−0.12^**^	−0.03	0.14^**^	1	
7. Online learning satisfaction	2.93 ± 0.92	−0.39^**^	−0.41^**^	0.18^**^	0.26^**^	−0.1^**^	−0.16^**^	1

** *p < 0.01 (two-tailed test)*.

### The Relation Among Constructs Studied at the Individual Level

The hierarchical linear regression analysis (see Table 2) showed that COVID-19-related stressors were positively related to symptoms of anxiety (*B* = 0.76, *p* < 0.001) and depression (*B* = 0.98, *p* < 0.001) and negatively related to perceived general health (*B* = −0.07, *p* < 0.05). Both problem-based coping and online learning satisfaction were negatively correlated with symptoms of anxiety and depression and positively correlated with perceived general health, while emotion-based coping was positively related to symptoms of anxiety and depression (all *p*-values < 0.05).

**Table 2:** Hierarchical linear analysis of the studied constructs among Chinese adolescents during the pandemic.

	**Anxiety**		**Depression**		**Perceived general health**	
	***B(SD)***	**Δ *R*^**2**^**	***B(SD)***	**Δ *R*^**2**^**	***B(SD)***	**Δ *R*^**2**^**
**Control variable**		0.02		0.03		0.01
Sex	1.04(0.37)^b^		1.43(0.47)^b^		−0.15(0.07)^a^	
Social status	−0.16(0.06)^b^		−0.24(0.06)^c^		0.001(0.01)	
**Predictors**		0.52		0.51		0.05
Online learning satisfaction	−1.3(0.14)^c^		−1.56(0.21)^c^		0.12(0.03)^c^	
COVID-related stressors	0.76(0.19)^c^		0.98(0.22)^c^		−0.07(0.03)^a^	
Problem-based coping	−0.19(0.03)^c^		−0.28(0.03)^c^		0.02(0.005)^c^	
Emotion-based coping	0.24(0.04) ^c^		0.34(0.04)^c^		−0.01(0.01)	
Problem-based coping^m^	−0.31(0.14)^a^		−0.16(0.16)		0.04(0.02)^a^	
Emotion-based coping^m^	0.51(0.19)^b^		0.93 (0.20)^c^		−0.06(0.03)^a^	
Online learning satisfaction^m^	−0.73(1.3)		−0.08(1.37)		−0.18(0.14)	
**Interaction terms**		0.02		0.02		0.01
Stress × pc	−0.39(0.17)^a^		−0.57(0.22)^b^		0.05(0.03)	
Stress × ec	0.55(0.16)^b^		0.52(0.19)^b^		−0.09(0.03)^b^	
Stress × learning	−0.32(0.19)		−0.36(0.18)^a^		0.01(0.04)	

a p < 0.05,

b p < 0.01,

c p < 0.001.

m *The mean score of variables based on class; Δ R^2^ the amount of total variance (both level 1 and level 2) in the dependent variable captured by added predictors in the model; stress, COVID-19-related stressors; PC, problem-based coping; EC, emotion-based coping; OLS, online learning satisfaction*.

**Figure 1:**
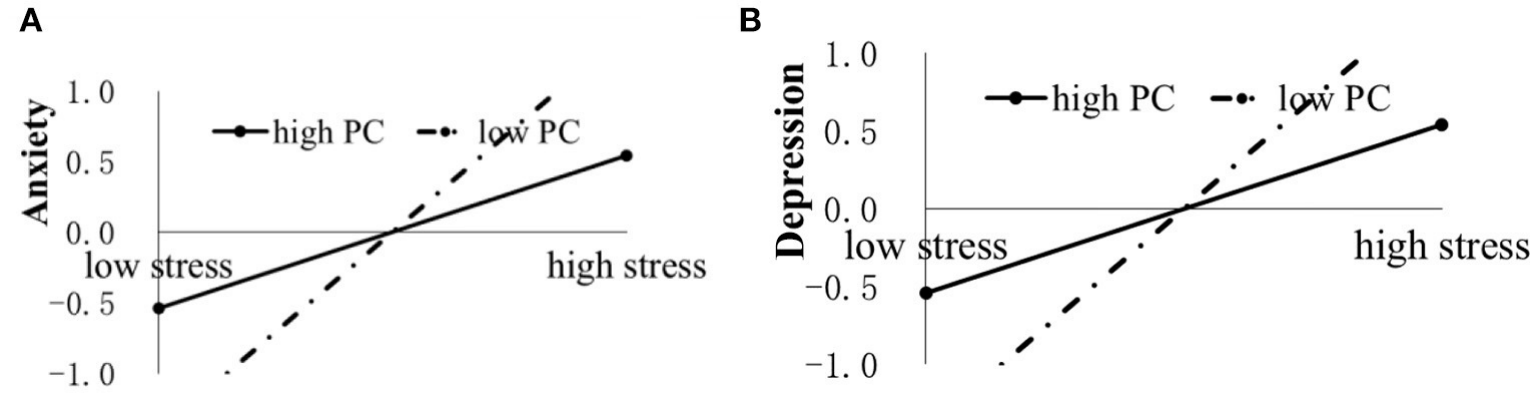
The impact of COVID-19-related stressors (stress) on anxiety **(A)** and depression **(B)** moderated by problem-based coping (PC).

**Figure 2:**
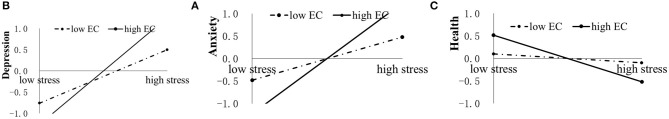
The impact of COVID-19-related stressors (stress) on anxiety **(A)**, depression **(B)**, and health **(C)** moderated by emotion-based coping (EC). Note: Health = perceived general health.

**Figure 3:**
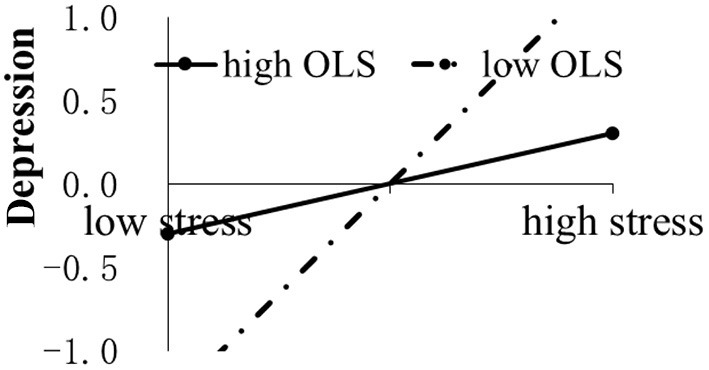
The impact of COVID-19-related stressors (stress) on depression moderated by online learning satisfaction (OLS).

Table 2 shows that the interaction between COVID-19-related stressors and coping or online learning satisfaction significantly predicted individual adjustment. For further interpretations, the interaction effects of two levels of coping or online learning satisfaction were plotted, with the low level defined as below−1 SD from the mean and the high level as above +1 SD from the mean. For problem-based coping, a simple slope analysis (22) showed that the relation between COVID-19-related stressors and psychological symptoms at a low level of problem-based coping was notably larger than that at a high level (for anxiety: β_low_ = 0.45, *p* < 0.001, β_high_ = 0.27, *p* < 0.01, *Z*(244) = 1.97, *p* < 0.05; for depression: β_low_ = 0.45, *p* < 0.001, β_high_ = 0.22, *p* < 0.05, *Z* (244) = 2.45, *p* < 0.01) (see Figures 1A,B). For emotion-based coping, the relation between COVID-19-related stressors and psychological symptoms at a high level of emotion-based coping was prominently larger than that at a low level (for anxiety: β_low_ = 0.24, *p* < 0.01, β_high_ = 0.38, *p* < 0.01, *Z*(244) = 1.99; for depression: β_low_ = 0.25, *p* < 0.01, β_high_ = 0.37, *p* < 0.01, *Z*(244) = 1.96) (see Figures 2A,B). In addition, the negative impact of COVID-19-related stressors on perceived general health was only found at a high level of emotion-based coping (β =−0.21, *p* < 0.05) but not at a low level (see Figure 2C). For online learning satisfaction, a positive correlation between COVID-19-related stressors and depression symptoms was found only at a low level of online learning satisfaction (β = 0.51, *p* < 0.001) and not at a high level (see Figure 3). Three levels of interaction among COVID-19-related stressors, coping, and online learning satisfaction were not found in the present study.

### The Relation Among the Studied Constructs at a Class Level

The results (see Table 2) showed that the mean scores of problem-based coping negatively predicted students' symptoms of anxiety (*B* = −0.31, *p* < 0.05) and were positively correlated with perceived general health (*B* = 0.04, *p* < 0.05), while emotion-based coping positively predicted symptoms of anxiety (*B* = 0.51, *p* < 0.01) and depression (*B* = 0.93, *p* < 0.001) and was negatively correlated with perceived general health (*B* = −0.06, *p* < 0.05). No interaction of individual-level variables and classroom-level variables could be found in predicting the adolescents' adjustment during the pandemic.

For the control variables, gender was positively correlated with symptoms of anxiety (*B* = 1.04, *p* < 0.01) and depression (*B* = 1.43, *p* < 0.01) and was negatively correlated with perceived general health (*B* = −0.15, *p* < 0.05), which indicated that female students reported more symptoms of anxiety and depression and poorer perceived general health than male students. Socio-economic status was negatively related to anxiety (*B* = −0.16, *p* < 0.01) and depression (*B* = −0.24, *p* < 0.001), which indicated that students with low socioeconomic status reported more symptoms of depression and anxiety.

## Discussion

To our knowledge, some studies have shown that adolescents can develop mental health problems due to the COVID-19 pandemic (2), but how mental health problems develop is not clear, which can influence the intervention effect on adolescents' adjustment during the pandemic. As few studies have investigated the impact of the COVID-19 pandemic on adolescents' adjustment by using cross-level analysis from the stress-health perspective, the findings of the present study enrich our understanding of the impact of the COVID-19 pandemic on the general adolescent population and are helpful in improving adolescents' adjustment during the pandemic.

### The Impact of Individual-Level Factors on Adolescents' Adjustment

At the individual level, the negative impact of COVID-19-related stressors on adolescents' adjustment is consistent with Main's view of the influence of infectious disease (e.g., SARS) on individual adjustment (8). This indicates that during an acute large-scale crisis such as the COVID-19 pandemic, even among individuals who are not directly infected with the disease, the psychological impact on the general adolescent population is significant. It expands the stressor-adjustment relationship from adults (e.g., college students) to adolescents during the pandemic, which can contribute to existing literature. Both problem-based coping and online learning satisfaction positively predicted individual adjustment. The positive impact of problem-based coping on adjustment is consistent with Lyon's findings on the buffer effect of problem-based coping in stress-health relations (9). This suggests that problem-based coping is adaptive during the pandemic and individuals can promote their adjustment by taking positive measures (e.g., take medical treatment) to alter the unfavorable environment around them. In the present study, the impact of emotion-based coping on adjustment is mixed. Specifically, emotion-based coping was found to be positively related to symptoms of anxiety and depression but not to perceived general health. The positive impact of emotion-based coping on psychological symptoms coincides with Main's findings during the SARS epidemic (8). This finding indicated that emotion-based coping is a negative coping style in promoting adjustment during infectious disease. The non-significant relationship of emotion-based coping and perceived general health indicates that the three indicators of adjustment (e.g., health, depression, anxiety) might have different antecedents during the pandemic. During a large-scale infectious disease, the symptoms of depression and anxiety were easily affected by major life events, while individual perceived general health was mainly influenced by individual physical health (e.g., illness). Therefore, compared with symptoms of depression and anxiety, individual perceived general health was not easily influenced by coping strategy including emotion-based coping (see the Δ*R*^2^ of three indicators of adjustment in Table 2).

The buffer effect of online learning satisfaction in the stress-adjustment relation was found in the present study. On the one hand, it supports the protective role of academic achievement (13). This suggests that adolescents who have high online learning satisfaction might be confident with their intelligence and tend to use active coping or have high efficacy in coping with stressors, which can reduce the negative impact of stressors on adjustment. On the other hand, adolescents might receive social support from their teachers and classmates in the process of completing online learning tasks, which can reduce the negative effect of stressors on adjustment. As few studies have investigated the impact of online courses on adolescents' adjustment, the present study contributes to the existing literature by investigating the buffering role of online learning satisfaction in the stress-health relationship during the pandemic.

### The Impact of Classroom-Level Factors on Adolescents' Adjustment

Most studies mainly focus on the influence of individual variables (e.g., coping) on adjustment during the pandemic, with less emphasis regarding the influence of class-level characteristics. The present study could provide more information about the influence of pandemic on adolescent's adjustment by analyzing the moderating role of coping at both individual and class levels. In the present study, the significant relation between the class-level indicator of coping and adjustment suggests that adolescents' adjustment is not only a function of students' own personal histories and expectations but also a result of classroom characteristics (16). The lack of interaction of stressors and class-level indicators of online learning satisfaction or coping in predicting adjustment suggests that although classroom climate is important in predicting individual adjustment, some COVID-19-related stressors (e.g., canceled vocational trips) are personal issues that are affected mainly by important people around the individual (e.g., parents) during the outbreak.

### The Impact of Demographic Variables on Adolescents' Adjustment

In terms of demographic variables, the results showed that female adolescents reported poorer adjustment than male adolescents during the pandemic. This finding is consistent with previous research (23, 24) and suggests that females tend to be more sensitive to external threats due to biological factors. For socioeconomic status, the findings are consistent with previous studies (25) and suggest that low socioeconomic status may be a potential threat to survival because impoverished adolescents have fewer resources for dealing with stressful events.

### Limitations

The present study has certain limitations. First, the sample bias should be considered when others interpret our findings because the sample used in the present study included only the adolescents from three secondary schools in mainland China. Second, it is a cross-sectional survey, which cannot make inferences about the causal relationships among studied variables. It is necessary to conduct further prospective and longitudinal studies to assess adolescents' adjustment and the predictors at different points within the context of COVID-19. Third, there has been some speculation that cultural differences exist between Eastern and Western adolescent samples in terms of interpreting or coping with stressful events (26, 27). Future research should consider cultural factors when discussing the relationships among the variables in the present study.

## Conclusions

The results of this study are an important part of a larger picture of intervention efforts in adolescents' adjustment during the COVID-19 pandemic. That is, COVID-19-related stressors were a vulnerability factor in predicting adjustment. Adjustment can be attributed to both individual-level (e.g., coping) and class-level (e.g., class-level indicators of coping) characteristics. Specifically, problem-based coping and online learning satisfaction can promote adolescents' adjustment directly or serve as a buffer against the negative impact of stressors on adjustment, while emotion-based coping is a vulnerability factor in predicting adjustment directly or serves as a risk factor in strengthening the relation between stressors and adjustment. Practices and strategies at school should focus on those factors in improving adolescents' adjustment during the pandemic.

## Implications for School Health

These findings have important implications for the design of health prevention programs for adolescents during the pandemic. The present study highlights several implications:

- The findings suggest that effective screening procedures should be developed to identify adolescents who experience many stressors and provide suitable psychological interventions for them.
- The findings also provide evidence that supports the implementation of strategies to reduce the negative impact of COVID-19-related stressors on adjustment. On the one hand, it suggests that psychologists and social workers could promote adolescents' adjustment by encouraging them to engage in problem-based coping. On the other hand, it suggests that school teachers could increase adolescents' online learning satisfaction by improving teaching quality, such as offering a high quality of learning materials and providing a convenient interactive question-and-answer platform.
- The findings suggest that secondary schools can promote adolescents' adjustment during the pandemic by constructing a comfortable class atmosphere.
- The findings suggest that schools should pay more attention to adolescents with low socioeconomic status because of their vulnerability to the pandemic.

## Data Availability Statement

The raw data supporting the conclusions of this article will be made available by the authors, without undue reservation.

## Ethics Statement

The studies involving human participants were reviewed and approved by the Research Ethics Committees of the School of Psychology, Jiangxi Normal University. Written informed consent to participate in this study was provided by the participants' legal guardian/next of kin.

## Author Contributions

XL wrote the manuscript, and all authors contributed to the article and approved the submitted version. All authors carried out the concepts, design, data acquisition, analysis, and manuscript editing.

## Conflict of Interest

The authors declare that the research was conducted in the absence of any commercial or financial relationships that could be construed as a potential conflict of interest.
